# A novel system for teaching the in-plane vascular access technique: A simulation study

**DOI:** 10.1097/MD.0000000000027201

**Published:** 2021-09-17

**Authors:** Mami Kikuchi, Takayuki Asao, Joho Tokumine, Alan Kawarai Lefor, Hisao Matsushima, Hideaki Andoh, Kazumi Tanaka, Masafumi Kanamoto, Yuki Ideno

**Affiliations:** a Center of Regional Medical Research and Education, Gunma University Hospital, 3-39-15 Maebashi, Gunma, Japan; b Gunma University Center for Mathematics and Data Science, 4-2 Aramaki, Maebashi, Gunma, Japan; c Department of Anesthesiology, Kyorin University School of Medicine, 6-20-2 Sinkawa, Mitaka, Tokyo, Japan; d Department of Surgery, Jichi Medical University, Shimotsuke, 3311-1 Yakushiji, Shimotsuke, Tochigi, Japan; e Emergency and Critical Care Center, Dokkyo Medical University Saitama Medical Center, 2-1-50 Minami Koshigaya, Koshigaya, Saitama, Japan; f Akita University Hospital Medical Simulation Center, 1-1-1 Hondou, Akita-City, Akita, Japan; g Medical Quality and Safety Management Center, Gunma University Hospital, 3-39-22 Maebashi, Gunma, Japan; h Intensive Care Unit, Gunma University Hospital, 3-39-15 Maebashi, Gunma, Japan.

**Keywords:** education, in-plane approach, simulation training, ultrasound guided central venous catheterization

## Abstract

The long-axis in-plane approach is amenable to ultrasound-guided central venous catheterization. However, the long-axis in-plane approach is considered difficult to learn because the needle should remain visible in the ultrasound beam during the procedure. We developed a novel competency-based modular system to acquire the skills for the long-axis in-plane approach. The purpose of this study is to evaluate the efficacy of this system.

The study was approved by the local ethics committee. Participants performed ultrasound guided venous catheterization (pre-test), attended a 2-hour hands-on session with the teaching system and were then evaluated again (posttest). The teaching system is a simulator device consisting of an ultrasound probe, a simulated vessel, a needle, and an endoscope connected to a computer to visualize the image inside the simulated vessel. The success rate, visualization of the needle tip, and puncture accuracy were measured before and after training. The puncture accuracy was determined by evaluating the distance of the needle tip and needle shaft from the center of a simulated vessel. Primary outcomes were the success rate and the puncture accuracy. The secondary outcome was needle tip visualization. McNemar test was used to analyze success rate and needle tip visualization. Tukey test was used to analyze puncture accuracy. A *P* value <.05 was considered statistically significant.

Forty-seven participants were enrolled in this study. The success rate was significantly increased (pre-test 79%, posttest 94%, *P* = .04). Ultrasound images from 42 participants were analyzed for puncture accuracy. Puncture accuracy significantly increased for needle tip distance (*P* = .03), but not shaft distance (*P* = .1). The needle tip visualization was significantly improved (*P* = .02).

A novel competency-based teaching system was constructed in a step-by-step manner, which improved needle tip visualization and puncture accuracy, with a higher success rate.

## 1 Background

Ultrasound guidance for vascular access is performed in several ways, including the short-axis out-of-plane, long-axis in-plane, and oblique approaches.^[1–3]^ These approaches take into consideration a direction perpendicular to the vessel axis and the relationship to the ultrasound beam. The basic skills are the out-of-plane and in-plane techniques. The out-of-plane technique is easy to learn but has the disadvantage of difficult needle tip recognition. The in-plane technique is difficult to learn but has the advantage of easy needle tip recognition.^[4,5]^ The reason for difficulty learning the in-plane technique is that the operator must keep the needle visible in the ultrasound beam during the procedure.

We developed a novel system to expedite acquiring the skills required for the in-plane technique. The learning system is structured in a step-by-step manner.

In this study, we evaluated the efficacy of this novel system for the long-axis in-plane approach by measuring the success rate, puncture accuracy, and needle tip visualization.

## 2 Methods

This study was approved by the local ethics committee (Gunma University Hospital Clinical Research Review Board, approved no. IRB1554). Participants were recruited by an advertisement for a hands-on seminar on the long-axis in-plane approach. The study was a single-group comparison using a pre-test and posttest design. Written informed consent was obtained from all participants. Exclusion criteria was experience in the in-plane technique for ultrasound-guided vascular access, and refusal to participate in the study.

### 2.1 Needle and ultrasound machine

Cannulas over needles (size; 22 G, length: 32 mm, Surflo I.V. catheter, Terumo Co., Tokyo, Japan) and ultrasound imaging (Isono, Alfabio Co., Japan) with a 10 MHz linear probe were used. The teaching system is a simulator device consisting of an ultrasound probe, a simulated vessel, a needle, and an endoscope connected to a computer to visualize the image inside the simulated vessel.

### 2.2 The novel training system

**Figure 1:**
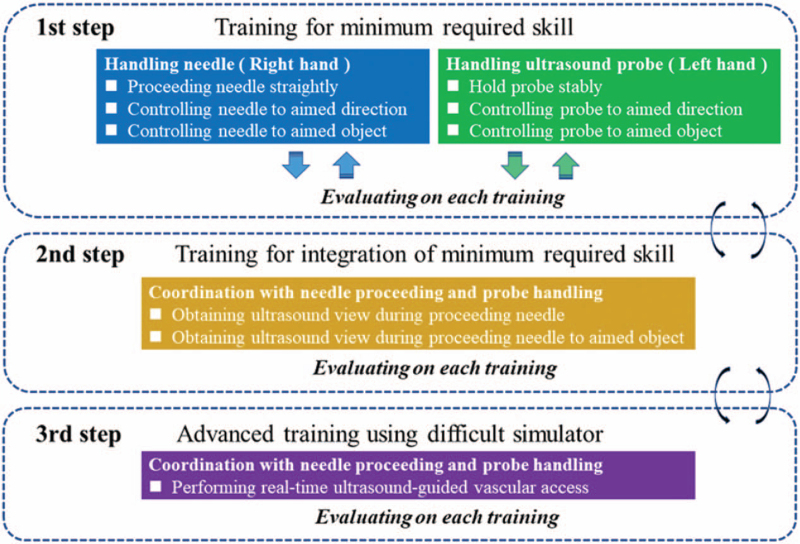
A novel competency-based modular teaching system. This system consists of 3 parts, which include the minimum required skills (1st step), integration of minimum required skills (2nd step), and advanced training (3rd step). The first step is training in basic skills, such as handling the needle and ultrasound probe. Handling the needle is composed of several fundamental maneuvers, including proceeding the needle straight, controlling the needle in the aimed direction, and controlling the needle toward the object aimed at. Hence, the trainee practices each component skill, and integrates them. Evaluation is conducted after integrating the component skills. If the trainee cannot pass the evaluation, they enter the feed-back loop training to acquire the necessary skill. Progression is based on acquiring competency in each component and not on time pent practicing the skill. The 1st step includes training for right and left hands. If the trainee acquires skill for both hands, the trainee then tries to acquire skill coordinating the motion of both hand (2nd step). In the 3rd step, trainees try several types of simulated vessels varying in depth, size, and direction.

The system includes competency-based feedback loops to enable learning the minimum components required for the complete skill set to perform the long-axis in-plane approach (Fig. 1).^[6]^ A characteristic of the learning system was that trainees could repeatedly practice each component skill until it is mastered, and then integrate them as a complete skill set.

### 2.3 Seminar and experiment^[7]^

**Figure 2:**
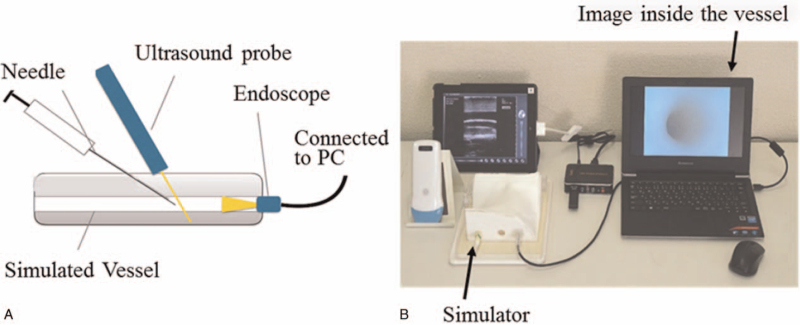
Recording system to obtain the endoscopic view inside the simulated vessel. The teaching system is a simulator device consisting of an ultrasound probe, a simulated vessel, a needle, and an endoscope connected to a computer to visualize the image inside the simulated vessel. Panel a: Endoscope is inserted into the simulated vessel. Panel b: Endoscopic and ultrasound views are recorded and saved in the computer simultaneously.

Before training, participants performed ultrasound-guided vascular puncture using a torso type simulator for the internal jugular vein (simulated vessel: diameter 6 mm, depth 10 mm) as the pre-test. Endoscopic and ultrasound images were recorded on a computer while participants performed puncturing the simulated vessel (Fig. 2). The obtained images were numbered to protect anonymity. Participants then attended a 2-hour lecture using the component-based learning system (Fig. 1). The training simulator was a box type simulator equipped with a simulated vessel (inner diameter 6 mm, depth 5 mm). After hands-on training, participants performed the posttest.

The ultrasound and endoscopic views were simultaneously recorded (Fig. 2). The recordings were sequentially numbered and the numbers later randomized by a computer to assure anonymity. The identification of individual participants was concealed for evaluation. Two ultrasonography experts, who did not participate in the seminars, observed the recordings and evaluated success rate, puncture accuracy, and needle tip visualization.

### 2.4 Success rate

Success was defined as the needle penetrating only the anterior vessel wall and the needle tip placed within the vessel. Failure occurred if the needle did not penetrate the vessel (non-puncture) or completely penetrated both anterior and posterior vessel walls (posterior wall penetration).

### 2.5 Puncture accuracy

**Figure 3:**
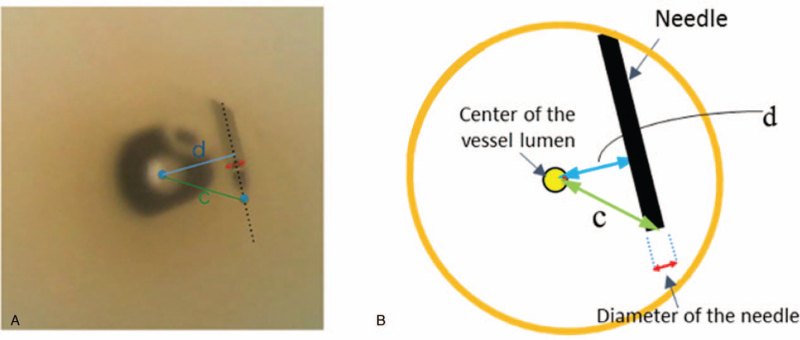
Puncture accuracy. Panel a: Recorded image, Panel b: Schematic diagram of the recorded image (panel a). Distance between the needle tip (a) or the needle shaft (d) from the center of the vessel lumen are measured on the recorded image (panel a). The magnification ratio was calculated from the measured needle diameter compared to the actual needle diameter. The actual distance between the needle tip or the needle shaft from the vessel center are calculated using the magnification ratio.

Puncture accuracy was evaluated by determining the distance from the needle tip or needle shaft to the center of the vessel (Fig. 3). The distance from the needle tip or needle shaft to the center of the vessel was measured on images captured at different magnifications. Actual distance was calculated with a magnification ratio.

### 2.6 Needle tip visualization

**Figure 4:**
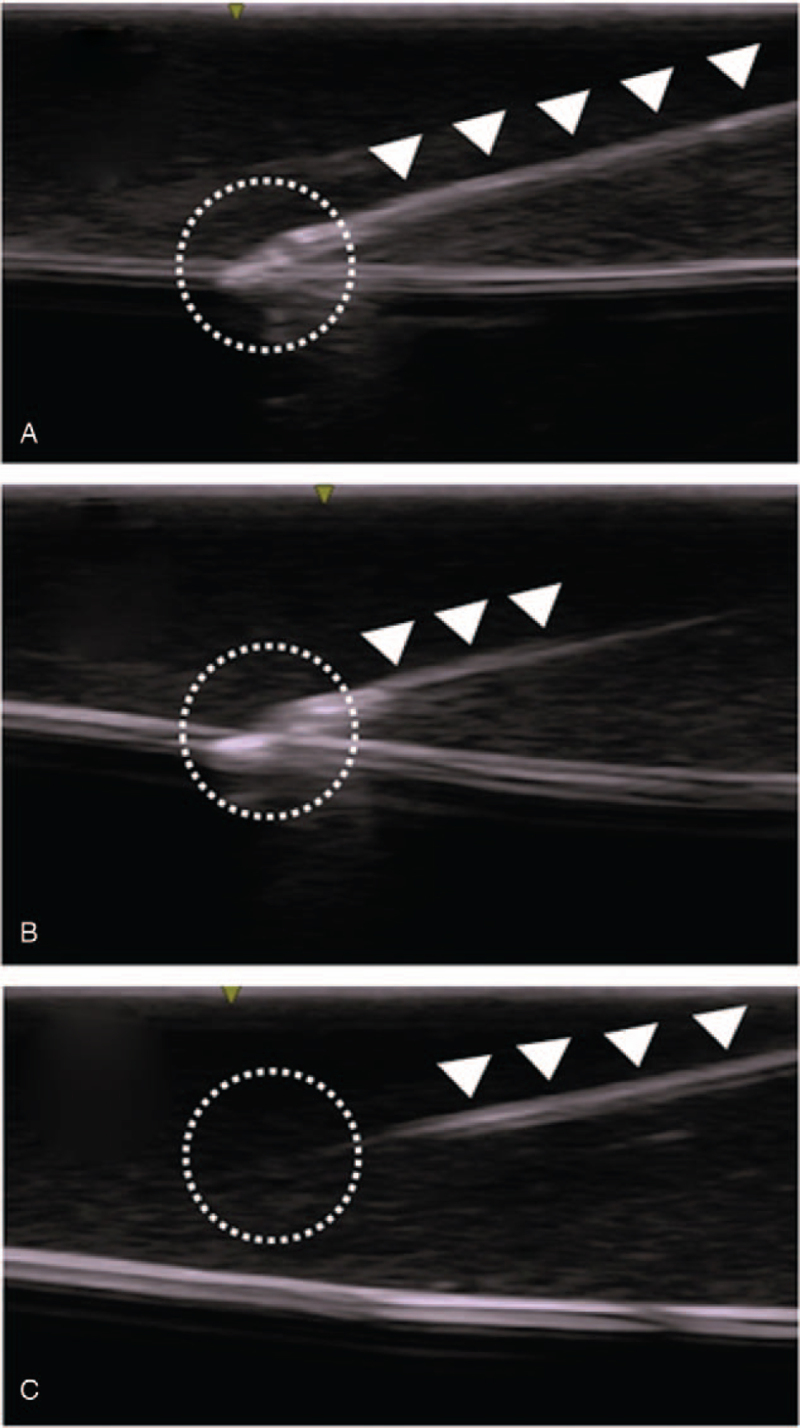
Needle tip visualization-representative images. Ultrasound experts observed the recorded ultrasound views and evaluated whether the needle tip could be classified as “identified” (clearly visible, or visible) or not (“unidentified”). Typical images judged as clearly visible, visible, and unidentified are shown. Panel a: Clearly visible: Needle tip and shaft are seen in full length. White closed triangles indicate the needle. Panel b: Visible: Needle tip and shaft are seen partially. Panel c: Unidentified: Needle tip cannot be observed. The shaft can be seen partially.

Needle tip visualization was evaluated based on visibility of the needle tip and shaft just before puncturing the simulated vessel (Fig. 4).

### 2.7 Statistical analysis

Sample size was calculated from a previous study using the same experimental setting.^[7]^ The sample size required for 80% power at ɑ = 0.05 was estimated to be 43 participants. Statistical analyses were performed using JMP Statistical Discovery^TM^ (ver. 12.2.2., SAS Institute Japan Ltd., Tokyo, Japan). McNemar test was used to evaluate the success rate and needle tip visualization. Tukey test was used for puncture accuracy. A *P* value <.05 was considered statistically significant.

In this study, primary outcomes were success rate and puncture accuracy. The secondary outcome was needle tip visualization.

## 3 Results

Six seminars to teach ultrasound-guided vascular access were conducted. Forty-seven participants were enrolled in the study including 15 senior physicians, 27 residents, and 5 nurses. Endoscopic images were obtained from all 47 participants. Their experience as health care providers varied (median 1 year, range from under 1 to 28 years). The number of previously performed ultrasound-guided central venous catheterizations were: senior physicians ≥ 30, residents 1–2, and nurses 0. Previous experience with ultrasound-guided central venous catheterization for all participants with experience used the short-axis out-of-plane approach. No participant had previous experience using the in-plane technique. All participants voluntarily completed the training.

Success rates significantly increased after simulation training (Table 1). Forty-two participants’ ultrasound images were analyzed for the puncture accuracy (15 senior doctors, 27 residents). Images from 5 participants could not be used for evaluation (1: unclear, 4: computer error). Puncture accuracy significantly increased when evaluated by needle tip distance, but not by shaft distance (Table 2). Needle tip visualization significantly improved after training (Table 3).

**Table 1:** Success and failure rates.

	Pre-test (%)	Posttest (%)
Success	37 (79%)	44 (94%)
Failure	10 (21%)	3 (6.4%)
Posterior wall puncture	6 (13%)	1 (2.1%)
Non-puncture	4 (8.5%)	2 (4.3%)
	*P *= .04	

**Table 2:** Puncture accuracy.

Distance from the vessel center (mm)	Pre-test(mean ± SD)	Posttest (mean ± SD)	*P* value
Needle tip	2.5 ± 1.5	2.0 ± 2.0	.03
Shaft	1.8 ± 1.6	1.4 ± 1.2	.10

**Table 3:** Needle tip visualization.

	Pre-test	Posttest
Identified	25 (60%)	35 (83%)
Cleary visible	14 (34%)	20 (47%)
Visible	11 (26%)	15 (36%)
Unidentified	17 (40%)	7 (17%)
	*P* = .03	

## 4 Discussion

In this study, a hands-on seminar for ultrasound-guided central venous catheterization using a novel modularized competency-based teaching system successfully facilitated acquiring the skills required for the in-plane technique. Ultrasound guidance is a useful tool, but skill is needed to take advantage of ultrasound guidance.

Simulation-based education for central venous catheterization has been shown to result in improved outcomes in clinical practice.^[8,9]^ However, most simulation studies were designed as time-based training. In time-based training, some trainees will acquire enough skill, but others cannot acquire sufficient skill to perform ultrasound-guided vascular access with its benefits. These trainees may need additional training and an elongated training schedule.

Several factors may influence the outcome of this study, such as whether participants routinely use ultrasound for vascular access. We did not conduct a subgroup analysis due the number of participants. Interestingly, success rates in the pre-test were different for senior physicians (100%), residents (70%), and nurses (60%), although all participants in this study were novices in using the long-axis in-plane technique. However, the puncture accuracy evaluated by measuring the distance from the needle tip to the center of the vessel was improved in the posttest compared to the pre-test. These results suggest that the background skill level of the learner may not affect the acquired skill which is the final goal of the learning system.

We also evaluated puncture accuracy by measuring the distance from the needle shaft to the center of the vessel, which was not different comparing pre-test and posttest results. Differences in the results of puncture accuracy may be related to physical characteristics of the long-axis in-plane technique. On the basis of ultrasound characteristics, the in-plane technique has the advantage of easily measuring the distance in anterior and posterior directions but has the disadvantage of not measuring the distance laterally.

McGraw et al^[10]^ developed a novel simulation-based curriculum for ultrasound-guided central venous catheterization. Their curriculum involved a structured program to obtain the skills required for ultrasound-guided central venous catheterization. They monitored participants during training using a hand motion analysis system. The fundamental idea of the learning strategy was similar to the strategy behind the system used in the present study. Their idea was based on precise evaluation, but education was based on teaching by instruction. Our system can be used as a self-learning tool and simultaneously allows acquisition of the skills needed to achieve proficiency in ultrasound-guided vascular access. Therefore, this novel system can overcome the disadvantages of time-based training seminar.

This teaching system can be used for e-learning by itself or it can be used as the content of a hands-on seminar as in this study. Participants in such a seminar can easily use it to review the material after the seminar and pursue advanced learning.

The basic skills for vascular access procedures are presented separately in this system, and each lesson has a staged goal, which can be set and achieved by the leaner themself. Hence, they can learn at their own individual pace anytime and anywhere. This system provides an environment for deliberate practice.^[11]^ The disadvantage is that real-time feedback from an instructor cannot be obtained when using this system for self-learning. Difficulty in maintaining motivation is an inherent drawback of all self-learning systems. However, for example, by using a web conferencing system, an instructor can receive video images of a learner's performance using a simple web camera and provide real-time feedback. Pape-Koehler et al^[12]^ reported that multimedia-based training improved surgical performance significantly. We believe that combining self-learning and interactive hands-on instruction will help improve learning effectiveness and maintain a learners’ motivation.

For participant skill evaluation, we used a smaller simulated vessel (inner diameter 6 mm) than the usual internal jugular vein in adult humans (inner diameter ≥ 10 mm). In clinical practice, the operator will encounter difficult venous access, including small, tortuous, collapsed, and vasospastic veins. To evaluate proficiency in ultrasound-guided vascular access, we used a small-sized simulated vessel to replicate difficult vascular access.

This study has acknowledged limitations. This study showed efficacy of a novel teaching system used during a hands-on seminar. However, the system was originally developed as a self-learning system. Hence, the efficacy of the system as a self-learning system must still be evaluated. The evaluation method corresponds to level 2b of Kirkpatrick model.^[13,14]^ Therefore, the clinical impact of the system is also undefined. The next step in this research is to determine whether the skills obtained through this learning system can influence a learner's sustained clinical behavior to improve patient outcomes.

The authors recommend using this novel system to acquire basic skills in vascular access before starting clinical activity. If a novice clinician has an insufficient clinical success rate, this novel system may be used to gain additional training to improve clinical skills and improve competency. Acquiring skills before performing vascular access procedures in a clinical setting may contribute to improved patient safety.

## 5 Conclusion

In this study, we showed significant improvement in success rate, puncture accuracy, and needle tip visualization using a novel competency-based modularized teaching system. We believe that this novel system will contribute toward patient safety by providing effective training to utilize the long-axis in-plane approach, which requires a refined skill set to perform successful ultrasound-guided vascular access.

## Author contributions

**Conceptualization:** Takayuki Asao.

**Data curation:** Mami Kikuchi, Kazumi Tanaka.

**Formal analysis:** Masafumi Kanamoto, Yuki Ideno.

**Investigation:** Mami Kikuchi, Takayuki Asao, Kazumi Tanaka.

**Project administration:** Joho Tokumine, Hisao Matsushima.

**Supervision:** Hideaki Andoh.

**Validation:** Hideaki Andoh.

**Writing – original draft:** Mami Kikuchi, Joho Tokumine.

**Writing – review & editing:** Alan Kawarai Lefor.
